# Valor del análisis de péptido natriurético en el diagnóstico y prevención de la insuficiencia cardíaca en poblaciones de alto riesgo

**DOI:** 10.1515/almed-2024-0122

**Published:** 2024-08-20

**Authors:** Damien Gruson

**Affiliations:** Departamento de Medicina de Laboratorio, Clínicas universitarias St-Luc and Université Catholique de Louvain, 10 Avenue Hippocrate, 1200 Bruselas, Bélgica; Centro de investigación en Endocrinología, Diabetes y Nutrición, Instituto de Investigación Clínica y Experimental, Clínicas universitarias St-Luc y UCLouvain, Bruselas, Bélgica; División de Tecnologías Emergentes de la Federación Internacional de Medicina de Laboratorio (IFCC), Milán, Italia

**Keywords:** insuficiencia cardíaca, diabetes, hipertensión, biomarcador, prevención, péptido natriurético

**Figura 1:**
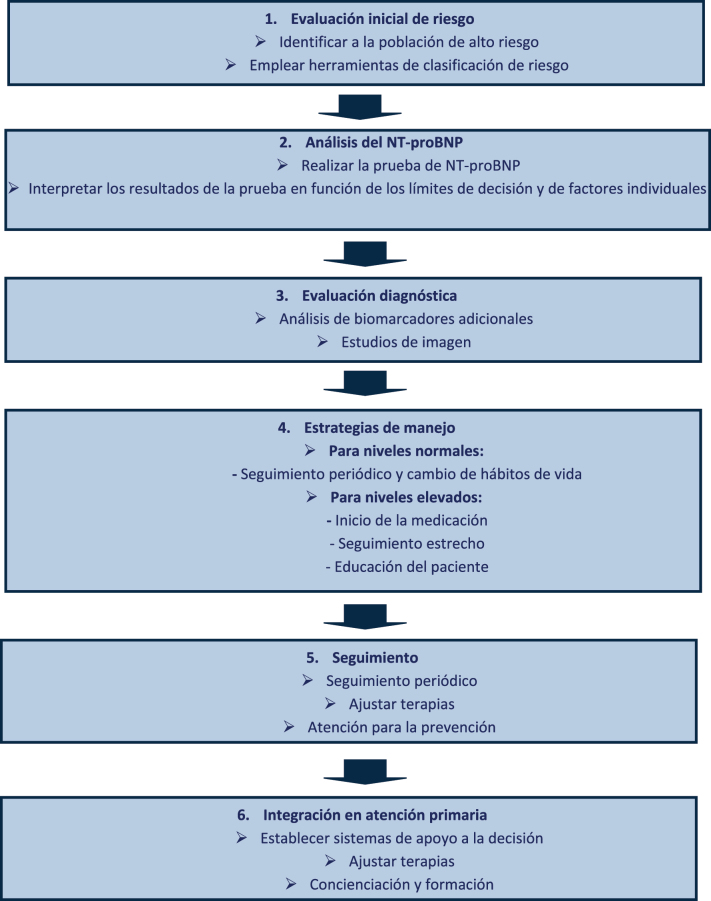
Detección y prevención de la insuficiencia cardíaca mediante el análisis de NT-proBNP en poblaciones de alto riesgo. Este diagrama de flujo ilustra el proceso a través del cual se emplea el análisis de NT-proBNP para la deteccion y prevención de la insuficiencia cardíaca en poblaciones de alto riesgo, que consiste en una evaluación inicial de riesgo, un análisis de NT-proBNP y las ulteriores estrategias de manejo en función de los resultados del análisis.

La insuficiencia cardíaca (IC) representa un desafío para los sistemas sanitarios de todo el mundo, especialmente en poblaciones de alto riesgo, como es el caso de los pacientes con diabetes mellitus, hipertensión y enfermedades cardiovasculares 1], [2], [3. El diagnóstico precoz, sumado a la prevención, resultan cruciales a la hora de mitigar la morbimortalidad asociada a la IC [1, 4, 5]. Cada vez existe mayor evidencia de la utilidad de los péptidos natriuréticos, particularmente el péptido natriurético tipo B (BNP) y su fragmento N-terminal (NT-proBNP), como biomarcadores en el diagnóstico y manejo de la IC [3]. En el presente editorial, analizamos la utilidad del análisis de péptidos natriuréticos en poblaciones de alto riesgo para el diagnóstico precoz y la prevención de la insuficiencia cardíaca (Figura 1).

Los péptidos natriuréticos son hormonas que se producen en el corazón como respuesta al aumento del volumen ventricular y la sobrecarga de presión. En la IC, los niveles de BNP y NT-proBNP aumentan significativamente, reflejando la gravedad de la patología [3]. Estos biomarcadores han sido ampliamente estudiados y validados para el diagnóstico, pronóstico y seguimiento terapéutico en la IC.

El diagnóstico precoz de la IC permite intervenir a tiempo y mejorar los resultados clínicos del paciente [2, 6]. El análisis de NT-proBNP es especialmente relevante en las poblaciones de alto riesgo. Por ejemplo, en pacientes con diabetes mellitus, una patología que incrementa significativamente el riesgo de desarrollar IC, el análisis de NT-proBNP puede ayudar a identificar la IC subclínica. Según un estudio de Lapi y col, la probabilidad de detectar precozmente la IC en pacientes diabéticos aumenta cuando se realiza el análisis de NT-proBNP [4]. Dicho estudio demostró que era significativamente más probable que el análisis de NT-proBNP se les prescribiera a pacientes con comorbilidades como cardiomiopatía isquémica, accidente cerebrovascular, fibrilación auricular e hipertensión, siendo todos ellos factores de riesgo asociados a la IC. La detección de poblaciones de alto riesgo de desarrollar IC mediante el análisis de NT-proBNP también puede ser de utilidad en las estrategias de prevención. Patel y col demostraron que el empleo del NT-proBNP en combinación con otras herramientas de detección como la clasificación de riesgo WATCH-DM permite identificar eficazmente a los individuos con riesgo elevado de desarrollar IC [5]. El estudio reveló que la prueba selectiva del NT-proBNP realizada en función de la clasificación WATCH-DM identificaba con eficacia a la población de prevención primaria con diabetes con alto riesgo de desarrollar IC que podría beneficiarse de terapias preventivas como la terapia con inhibidores del cotransportador de sodio y glucosa tipo 2 (SGLT2).

La utilidad clínica del NT-proBNP va más allá de su valor diagnóstico, ya que también aporta información pronóstica que puede resultar de utilidad en las decisiones terapéuticas y facilitar el seguimiento de la progresión de la enfermedad [3]. El costo efectividad del análisis de NT-proBNP se ha analizado en diversos contextos. Por ejemplo, en la estrategia de detección en dos pasos, el uso combinado del NT-proBNP con otros biomarcadores (p.ej. troponina cardíaca de alta sensibilidad) o modalidades de estudios de imagen (p.ej ecocardiografía) ha demostrado capturar una proporción significativa de los eventos de IC, manteniendo el coste de la detección a niveles razonables [1, 3].

La identificación temprana de la IC a través del análisis de péptidos natriuréticos permite intervenir de manera temprana con terapias que puedan ralentizar la progresión de la enfermedad, reducir las hospitalizaciones y mejorar la supervivencia. Se ha demostrado que medicaciones como los inhibidores del SGLT2 y los inhibidores de la neprilisina y del receptor de la angiotensina (ARNI) son eficaces a la hora de reducir la morbimortalidad relacionada con la IC. De este modo, la incorporación de las pruebas de péptidos natriuréticos en la práctica clínica rutinaria en atención primaria podría mejorar el manejo general de los pacientes de alto riesgo [1]. La realización del análisis de péptidos natriuréticos en atención primaria podría mejorar el manejo de las poblaciones de alto riesgo. Lapi y col subrayaron el potencial del NT-proBNP como herramienta de apoyo a la decisión en atención primaria, especialmente en pacientes con diabetes [4]. Los autores recomiendan desarrollar sistemas de apoyo a la toma de decisiones, para ayudar a los médicos de atención primaria a identificar a los pacientes que se podrían beneficiar de las pruebas de NT-proBNP y el ulterior manejo de la IC. La figura contiene un diagrama de flujo para el empleo del análisis de NT-proBNP en la detección precoz de la IC en poblaciones de alto riesgo.

En conclusión, el análisis de péptido natriurético desempeña un papel fundamental en el diagnóstico precoz y la prevención de la insuficiencia cardíaca en poblaciones de alto riesgo. Su uso en la práctica clínica podría facilitar la intervención temprana, mejorar los resultados clínicos de los pacientes, y contribuir a una asistencia sanitaria sostenible. A la luz de la creciente evidencia científica disponible respaldando la integración del análisis de péptido natriurético en los protocolos diagnósticos y de detección, los facultativos deberían plantearse adoptar estas estrategias, con el fin de mejorar la atención a los pacientes con riesgo elevado de padecer IC.
